# Exploration of Novel Xanthine Oxidase Inhibitors Based on 1,6-Dihydropyrimidine-5-Carboxylic Acids by an Integrated in Silico Study

**DOI:** 10.3390/ijms22158122

**Published:** 2021-07-29

**Authors:** Na Zhai, Chenchen Wang, Fengshou Wu, Liwei Xiong, Xiaogang Luo, Xiulian Ju, Genyan Liu

**Affiliations:** 1 Hubei Key Laboratory of Novel Reactor and Green Chemical Technology, School of Chemical Engineering and Pharmacy, Wuhan Institute of Technology, Wuhan 430205, China; zhainaaurora@163.com (N.Z.); Cheryl_CiCi@163.com (C.W.); fswu@wit.edu.cn (F.W.); xgluo@wit.edu.cn (X.L.); Xiulianju2001@yahoo.com (X.J.); 2 Hubei Key Laboratory of Plasma Chemistry and Advanced Materials, Wuhan Institute of Technology, Wuhan 430205, China; 3 School of Materials Science and Engineering, Zhengzhou University, No. 100 Science Avenue, Zhengzhou 450001, China

**Keywords:** xanthine oxidase inhibitor, 3D-QSAR, docking, pharmacophore, virtual screening

## Abstract

Xanthine oxidase (XO) is an important target for the effective treatment of hyperuricemia-associated diseases. A series of novel 2-substituted 6-oxo-1,6-dihydropyrimidine-5-carboxylic acids (ODCs) as XO inhibitors (XOIs) with remarkable activities have been reported recently. To better understand the key pharmacological characteristics of these XOIs and explore more hit compounds, in the present study, the three-dimensional quantitative structure–activity relationship (3D-QSAR), molecular docking, pharmacophore modeling, and molecular dynamics (MD) studies were performed on 46 ODCs. The constructed 3D-QSAR models exhibited reliable predictability with satisfactory validation parameters, including q^2^ = 0.897, R^2^ = 0.983, r_pred_^2^ = 0.948 in a CoMFA model, and q^2^ = 0.922, R^2^ = 0.990, r_pred_^2^ = 0.840 in a CoMSIA model. Docking and MD simulations further gave insights into the binding modes of these ODCs with the XO protein. The results indicated that key residues Glu802, Arg880, Asn768, Thr1010, Phe914, and Phe1009 could interact with ODCs by hydrogen bonds, π-π stackings, or hydrophobic interactions, which might be significant for the activity of these XOIs. Four potential hits were virtually screened out using the constructed pharmacophore model in combination with molecular dockings and ADME predictions. The four hits were also found to be relatively stable in the binding pocket by MD simulations. The results in this study might provide effective information for the design and development of novel XOIs.

## 1. Introduction

Gout is a clinical syndrome accompanied by some chronic and recurrent symptoms, such as pain, inflammation, and swelling [1,2,3,4]. Such an unhealthy condition originates from the superabundant presence of serum uric acid (SUA), which has been defined as hyperuricemia (HUA) and regarded as the primary cause of gout [5,6,7]. In the last decade, the dramatic increase of HUA patients has driven HUA to be the second metabolic disease following by the type II diabetes [8]. Uric acid (UA) and reactive oxygen species (ROS), as the oxidative end-products of xanthine and hypoxanthine, are generated in the purine scavenging pathway under the catalysis of xanthine oxidase (XO) [9,10]. The excess UA and ROS usually associate with many pathogeneses, such as inflammation, atherosclerosis, and carcinogenesis [11,12,13]. Clinical evidence has indicated that XO was a promising target for effectively treating with several diseases, especially with the HUA-associated conditions [8,9,10,11,12,13,14].

**Figure 1:**
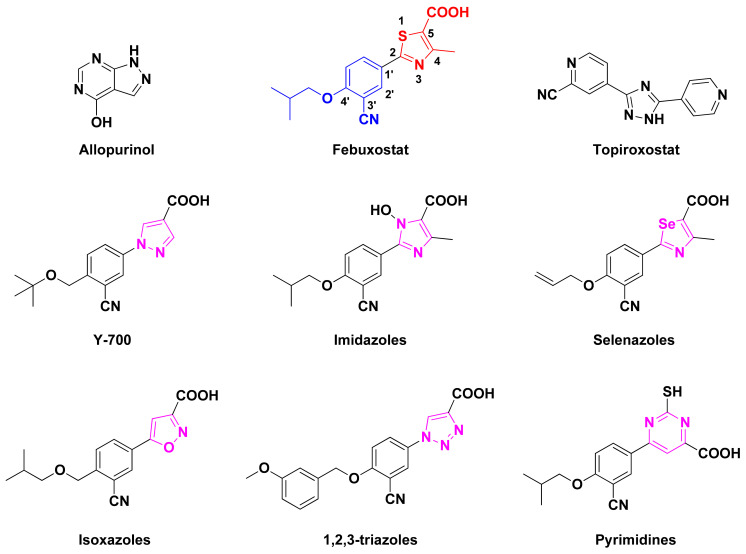
Chemical structures of different XOIs. Febuxostat contains an aromatic moiety (blue) and a nitrogen heterocyclic ring (red). Other febuxostat analogues have different nitrogen-containing heterocyclic rings (magenta).

In the past few decades, XO inhibitors (XOIs) have been widely used in the clinical treatment of HUA. Allopurinol, as a XOI pioneer, was discovered for HUA treatment in the 1960s [15]. However, allopurinol and its analogues with the semblable purine backbone have been restrained to use in some cases, owing to severe and life-threatening side effects like fever, rashes, kidney toxicity, and Stevens–Johnsons syndrome [16,17,18]. Febuxostat and topiroxostat, as novel and effective non-purine XOIs, were approved for marketing in 2009 and 2013, respectively [19,20]. However, these drugs are not regarded as a precise and safe therapy because of their untoward effects on patients, which has prompted researchers to explore and develop novel XOIs with alternate scaffolds and fewer adverse reactions [21,22,23,24]. Various chemotypes of non-purine XOIs have been reported in recent years, such as benzoflavone derivatives [20], 2-aryl/heteroaryl-4-quinolones [11], and 1-hydroxy-2-phenyl-4-pyridyl-1H-imidazoles [18]. In addition, the replacement of the thiazole ring of febuxostat with other heterocyclic rings (magenta, Figure 1), such as pyrazole (e.g., Y-700) [7], imidazole [18], triazole [9], isoxazole [12], thiazole [19], and selenazole [5], could produce more alternatives for novel XOIs.

As shown in Figure 1, febuxostat and its analogues interact with XO through two key pharmacophores, including an aromatic moiety (blue) and a nitrogen-containing heterocyclic ring (red) [6,14]. The linker of two pharmacophores could be modified by the extension of carbon chain or the introduction of heteroatoms like nitrogen. The carboxylate moiety of the nitrogen-containing heterocyclic ring (red) in febuxostat and its analogues act as the most tightly binding part of XOIs with the protein [15,23]. It has been confirmed that the presence of an electron-withdrawing substituent at the 3′ position of the febuxostat, such as a cyano or a nitro, resulted in an increase of inhibition efficiency [5,7]. The tetrazole ring could serve as a hydrogen-bond acceptor (HBA) to participate in forming privileged interactions with some residues in the XO binding pocket [13]. Recently, a novel series of febuxostat analogues, 2-substituted 6-oxo-1,6-dihydropyrimidine-5-carboxylic acids (ODCs), have been designed and synthesized based on bioisosteric replacement and ring enlargement strategies [3,4]. Some of these analogues exhibited better inhibitory activities than febuxostat against the XO protein in vitro.

To explore the structure–activity relationships (SARs) of these novel ODC XOIs and find more ideal candidates, in this study, 46 ODCs were selected for the integrated modeling studies. Three-dimensional quantitative SAR (3D-QSAR) models, including comparative molecular field analysis (CoMFA) and comparative molecular similarity indices analysis (CoMSIA), and pharmacophore models, were constructed using these ODCs. Meanwhile, molecular dockings were served to obtain the possible binding conformations of these XOIs and to explore their action mechanism. To explore novel XOI scaffolds, virtual screening was then carried out by pharmacophore model; molecular dockings; and predictions of absorption, distribution, metabolism, and excretion (ADME). Molecular dynamics (MD) simulations were subsequently performed to verify the rationality of the docking method, and to further analyze the effects and stability of hit compounds in the XO binding pocket. This study might provide important information and more alternatives for the design and development of novel XOIs.

## 2. Results and Discussion

### 2.1. CoMFA and CoMSIA Statistical Results

A dataset of 46 ODCs was selected for 3D-QSAR modelling, and their structures and biological activities represented by half-maximal inhibitory concentrations (IC_50_s) were given in Table 1. The internal and external validation parameters of the CoMFA and CoMSIA models were summarized in Table 2 and Table 3, respectively, using the same training (35 molecules) and test (11 molecules) sets. The CoMFA model gave rational parameters with a cross-validation correlation coefficient (q^2^) of 0.897, an optimum number of components (ONC) of 7, a standard error of estimate (SEE) of 0.050, a non-cross-validated correlation coefficient (R^2^) of 0.983, an F-statistic values (F) of 229.50, and a predictive correlation coefficient (r_pred_^2^) of 0.948 in the partial least squares (PLS) analysis. The external validation parameters of the CoMFA model, including a root-mean-squared error (RMSE) of 0.074, a Δr_m_^2^ of 0.052, and a $\bar{r_{m}^{2}}$ of 0.864, were also considered to meet the requirements. The contributions of steric and electrostatic fields were 77.3% and 22.7%, respectively.

Different combination modes of steric, electrostatic, hydrophobic, hydrogen-bond donor (HBD), and HBA fields were used to construct different CoMSIA models, and the q^2^ values of all possible combinations were summarized in Figure S1. The statistical parameters of the six best CoMSIA models were shown in Table 2. Eventually, the CoMSIA-SEHDA model was chosen as the optimal predictive CoMSIA model for the further analyses. As for the CoMSIA-SEHDA model, the q^2^, SEE, R^2^, F, r_pred_^2^, RMSE, Δr_m_^2^, and $\bar{r_{m}^{2}}$ were 0.922, 0.041, 0.990, 212.26, 0.840, 0.130, 0.118, and 0.717, respectively. The contributions of steric, electrostatic, hydrophobic, HBD, and HBA fields were 10.5%, 24.8%, 37.2%, 19.3%, and 8.2%, respectively. All above statistical parameters indicated that the constructed CoMFA and CoMSIA models could be used for the following study, and the electrostatic, hydrophobic, and HBD fields might be significant for the improvement of ODCs activity.

**Figure 2:**
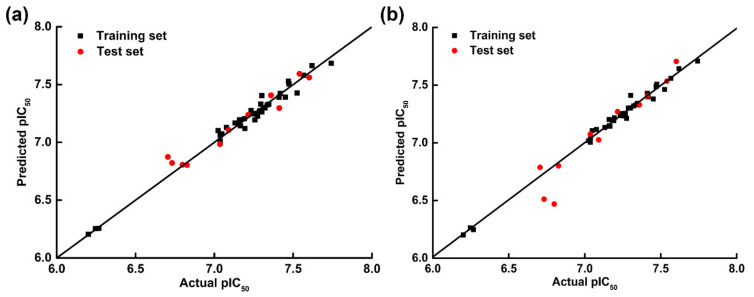
Scatter plots of actual versus predicted pIC_50_ values for the used XOIs based on the CoMFA (**a**) and CoMSIA-SEHDA (**b**) models.

The obtained CoMFA and CoMSIA models were then applied to predict the bioactivities of the training and test compounds. The actual pIC_50_s (−logIC_50_), predicted pIC_50_s, and their residuals were listed in Table 1. All the residuals were smaller than 0.4, suggesting that the CoMFA and CoMSIA models exhibited good predictivity. To further exhibit the relationships between the actual and predicted activities of all compounds, the scatter plots were depicted in Figure 2. As shown in Figure 2, the two outlier points were related to compounds **41** and **42**, whose predicted activities based on the CoMSIA model were slightly lower than their actual activity. All residual values (**41**: 0.2199; **42**: 0.3296) were in the reasonable range. The statistic points of other compounds exhibited great linear correlation, indicating that the 3D-QSAR models possessed high quality for the activity prediction of ODCs.

### 2.2. Contour Maps of the CoMFA and CoMSIA Models

**Figure 3:**
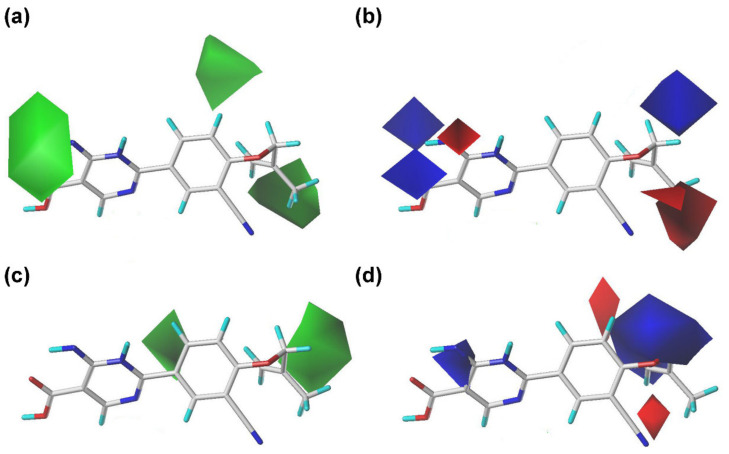
Contour maps of the CoMFA and CoMSIA-SEHDA models using compound **44** as a reference. (**a**) The steric contours of the CoMFA model. (**b**) The electrostatic contours of the CoMFA model. (**c**) The steric contours of the CoMSIA model. (**d**) The electrostatic contours of the CoMSIA model.

**Figure 4:**
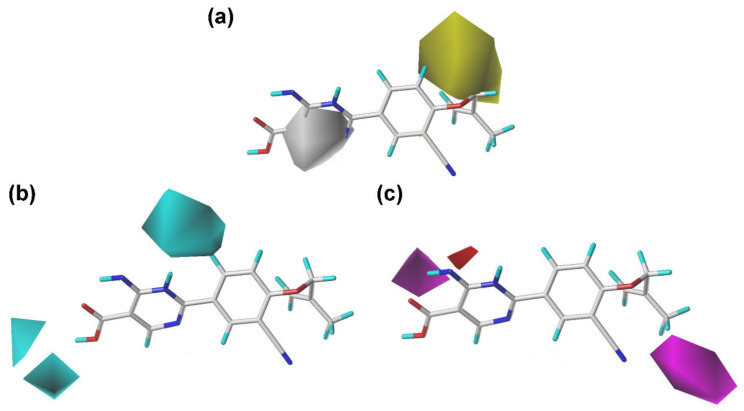
Contour maps of the CoMSIA-SEHDA model using compound **44** as a reference. (**a**) The hydrophobic field. (**b**) The hydrogen-bond donor field. (**c**) The hydrogen-bond acceptor field.

The CoMFA and CoMSIA contour maps with the most potent compound **44** as a reference molecule were shown in Figure 3 and Figure 4, respectively. As shown in Figure 3, the sterically advantageous and disadvantageous contours were colored in green and yellow, respectively. A medium green contour surrounding the R_1_ position of compound **44** in both CoMFA and CoMSIA models indicated that bulky substituents at this position might be beneficial to the activity. This was supported by the activity orders as follows: **29** (*iso*-pentyl) > **28** (*iso*-butyl) > **27** (*iso*-propyl) and **4** (*iso*-pentyl) > **3** (*iso*-butyl) > **2** (*iso*-propyl). Another small green contour neared the *meta*-position of the phenyl ring (R_1_) in the CoMFA and CoMSIA models, demonstrating that big substituents for structural modification at this place might be favorable for the increment of activity. In the steric contours of the CoMFA model, a large green contour covering the R_3_ position of the pyrimidine ring highlighted the importance of large groups in this region. The fact that an imino substituent at the R_3_ position was better for the activity than an oxygen atom could explain this result, as illustrated in the activity orders: **43** (R_3_ = NH) > **32** (R_3_ = O) and **44** (R_3_ = NH) > **31** (R_3_ = O).

The electrostatic maps of the CoMFA and CoMSIA models were shown in Figure 3; the blue contours denoted that electropositive groups were favor in these regions, whereas the red contours were the opposite. It could be observed that a medium blue contour in the CoMFA model and a large blue contour in the CoMSIA model lied in the R_1_ position, which were congruent with the following activity order: **5** (allyl) > **8** (propinyl) and **4** (*iso*-pentyl) > **7** (*iso*-pentenyl), attributed to the existence of electropositive substituents. A medium red contour in the CoMFA model and a small red contour in the CoMSIA model appeared over the *pata*- or *meta*-position of the phenyl ring (R_1_), indicating that the occupation of electronegative groups at this region was advantageous for increasing the inhibitory activity. This result was in accordance with the experimental data: **20** (*m*-fluorobenzyl) > **12** (benzyl) and **36** (*p*-bromobenzyl) > **37** (*p*-tert-butylbenzyl). Two small blue contours in the CoMFA model and one small blue contour in the CoMSIA model were located near the pyrimidine ring, which were in accordance with the actual activity orders: **45** (R_3_ = NH) > **36** (R_3_ = O) and **46** (R_3_ = NH) > **38** (R_3_ = O), as the electropositivity of the oxygen is weaker than that of the nitrogen.

Regarding the hydrophobic contours of the CoMSIA model (Figure 4a), the yellow and white contours represented favor and unfavored regions for the hydrophobic groups, respectively. The existence of a big yellow contour at the R_1_ position revealed that the substituents of hydrophobic groups in this position tended to increase the activity, such as the following activity orders: **13** (*p*-methylbenzyl) > **12** (benzyl), **38** (*p*-methylbenzyl) > **33** (benzyl), and **1** (methyl) > **26** (H). There was a white contour covering the R_2_ position of the pyrimidine ring, suggesting that the hydrophobic groups in this position might not be beneficial for the activity improvement. For instance, compounds **29**, **31**, **36**, and **38** without substituent at the R_2_ position exhibited higher activity than the corresponding compounds **39**, **40**, **41**, and **42** with a methyl group, respectively.

The HBD and HBA contour maps of the CoMSIA model are shown in Figure 4. For the HBD contours, the cyan contours suggested HBD groups were advantageous for the activity at the corresponding regions. For the HBA contours, the magenta regions represented that the occupation of HBA substituents surrounding these regions might facilitate the bioactivity improvement, whereas the red contours mean the contrary effect. Two small cyan contours and a medium-sized magenta contour were observed over the carboxyl group of the pyrimidine ring in the HBD and HBA contours, respectively. These observations were consistent with a previous study that the carboxylate group of febuxostat analogues was essential for the inhibitory activity since it could serve as HBDs or HBAs to form critical hydrogen bonds with the XO protein [22]. Besides, there was another medium cyan contour near the pyrimidine ring in the HBD contours, suggesting that the nitrogen atoms of the pyrimidine ring might serve as HBDs to interact with the protein. One medium magenta was observed near the benzene ring of the common skeleton in the HBA contour map. This manifested that the HBA existence in the common scaffold, such as a tetrazole ring or a cyano group, might be important for the inhibitory activity.

According to the analyses of the 3D-QSAR models, the appropriate substituents for improving the inhibitory activity of these ODC XOIs at specific positions might be concluded as follows: (1) bulky, positive charged, and/or hydrophobic groups at the R_1_ position; (2) negative charged groups at the *para*- or *meta*-position of the phenyl ring at the R_1_ position; (3) hydrophilic groups at the R_2_ position; (4) bulky and/or positive charged groups at the R_3_ position; (5) a tetrazole ring or a cyano group at the phenyl ring as HBAs; (6) a carboxyl group of the pyrimidine ring as HBDs or HBAs; and (7) the nitrogen atom of the pyrimidine ring as HBDs.

### 2.3. Molecular Docking

**Figure 5:**
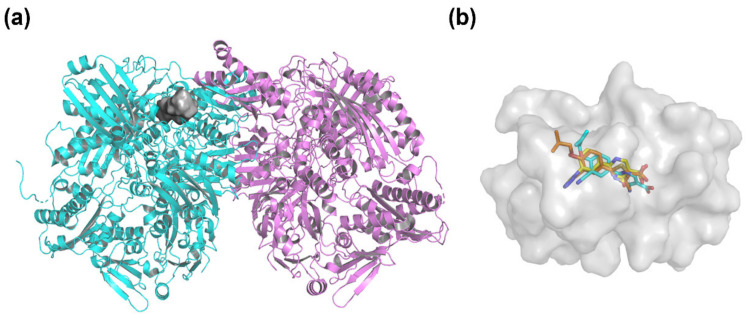
The binding pocket of the XO protein (PDB ID: 1N5X). (**a**) The binding site of the XO protein (chain A, cyan cartoon; chain B, violet cartoon; binding pocket, gray surface). (**b**) The comparisons of febuxostat (orange sticks), compound **44** (cyan sticks), and compound **26** (yellow sticks) in the docking position of the binding pocket (gray surface).

**Figure 6:**
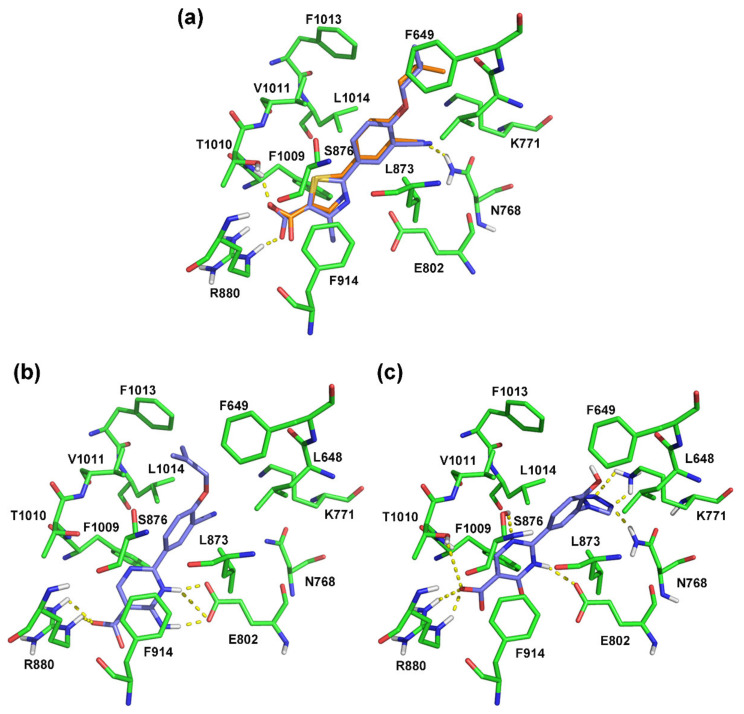
The docking results of febuxostat (**a**), compound **44** (**b**), and compound **26** (**c**) in the binding site of protein (PDB ID: 1N5X). The crystal ligand, redocked ligand, important residues, and hydrogen bond were shown in orange sticks, blue sticks, green sticks, and yellow dashes, respectively.

To validate the reliability of the docking method and explore the key interactions, the co-crystallized febuxostat was extracted and then redocked into the XO protein (PDB ID: 1N5X). As shown in Figure 5a, the XO protein was a homodimer, in which one of subunit (blue cartoon) was selected to generate binding pocket (highlighted as gray surface) by the surflex-docking method. Febuxostat and 46 ODCs were successively docked, and their docking scores were summarized in Table S1. The docking superimposition of compounds febuxostat, **44**, and **26** was highlighted in Figure 5b to compare their differences in the binding positions. As shown in Figure 6a, the conformation of the redocked febuxostat almost completely overlapped with that of the crystal ligand, with a root-mean-square deviation (RMSD) value of 0.867 Å (<2 Å) and a docking score of 8.22. This indicated that the used docking method and the related parameters were reasonable. The carboxylate group of febuxostat formed hydrogen bonds with Arg880 (Arg880-N-H…O=C, 2.1 Å) and Thr1010 (Thr1010-N-H…O=C, 2.0 Å), and the cyano group interacted with Asn768 by a hydrogen bond (Asn768-N-H…N≡C, 1.9 Å). These hydrogen bonds might be significant for maintaining the binding conformation of febuxostat. The phenyl ring embedded into a hydrophobic pocket that was generated by the surrounding amino acid residues Leu648, Phe649, Leu873, Val1011, Phe1013, Leu1014, Phe914, and Phe1009. Moreover, the π-π stacking was also found between the thiazole ring of febuxostat and the phenyl groups of the aromatic residues Phe914 (face-to-face) and Phe1009 (face-to-edge). These interactions were also important for the binding of febuxostat to the XO protein, as reported in a previous study [12,23]. The above-mentioned results indicated that the docking method was reliable and could be used for the following experiments.

Forty-six ODC XOIs (Table 1) were then docked into the binding site using the same pattern. The docking modes of the most active compound **44** and the least active compound **26** are shown in Figure 6. The docking score of compound **44** (10.69) was much higher than that of compound **26** (7.85), which was in agreement with their experimental activities. In general, we found that the docking score order of these XOIs was accordant with their inhibition potencies. Moreover, it was noted that compounds febuxostat, **44**, and **26** have similar docking orientations and binding interactions in the pocket. Similar to febuxostat, compound **44** (Figure 6b) formed hydrogen bonds with Arg880 (Arg880-N-H…O=C, 2.4 Å; Arg880-N-H…O=C, 2.2 Å) by the carboxylate group. The additional hydrogen bonds were formed between Glu802 and the nitrogen atom of the pyrimidine ring (Glu802-C=O…H-N-C, 1.9 Å; Glu802-C=O…H-N-C, 2.6 Å; Glu802-C=O…H-N=C, 1.9 Å).

The docking result of compound **26** (Figure 6c) showed that the carboxylate group, the nitrogen atom of the pyrimidine ring, and the tetrazole group were generated the semblable hydrogen bonds with Arg880 (Arg880-N-H…O=C, 2.3 Å; Arg880-N-H…O=C, 1.8 Å), Thr1010 (Thr1010-C-O-H…O=C, 2.5 Å), Glu802 (Glu802-C=O…H-N-C, 2.1 Å), and Asn768 (Asn768-N-H…N=C, 1.9 Å), respectively. Furthermore, it could be observed that some hydrogen bonds were formed between the nitrogen atom of the pyrimidine ring and Ser876 (Ser876-O-H…N=C, 2.6 Å), and the nitrogen atoms of the tetrazole group and Lys771 (Lys771-N-H…N=C, 2.4 Å; Lys771-N-H…N=C, 2.0 Å).

Compared to the conformation of febuxostat, the changes in the docking results of compound **44** might be caused by the deeper binding position and the rotation of the benzene ring. These variations of compound **44** might have caused the purine ring to form a hydrogen bond with Glu802. The previous study indicated that Glu802 as a key residue could form at least one hydrogen bond with the amino group or the nitrogen-containing heterocyclic ring of febuxostat analogues [8,15]. In comparison with compound **44**, compound **26** was found to loss partial interactions with Glu802, which might be considered as the primary reason caused its decreased potency.

An online web service Protein Contacts Atlas (http://www.mrc-lmb.cam.ac.uk/pca/ (accessed on September 2020)) was also used for validating key residues interacted with the ligand febuxostat in XO protein [25]. It could be seen from Figure S2 that there were an inner circle and an outer circle, representing directly and indirectly interacted residues with febuxostat (central blue note), respectively [26]. The circle size of each residue represented the number of atomic contacts. It is worth noting that Phe914, Glu802, Phe1009, Arg880, Leu648, and Thr1010 might play important roles for febuxostat binding, consistent with the docking results.

### 2.4. Pharmacophore Model

The pharmacophore models were built using different selections of 12 compounds, and the partial modeling results were displayed in Table S2. In order to make the pharmacophores more reasonable, febuxostat, which has structural similarity with ODCs, was given preference to construct models. The selection of remaining 11 molecules was followed the principles of relatively higher structural diversity and better activity. Twenty pharmacophore models were established by aligning and comparing the common features from a set of 12 active compounds (Table 1), and their statistical results were listed in Table 4. By comparing potential models based on different selections, the FEATS values were almost unaffected. If the modeling molecules possessed too high structural difference, it might cause poor N_HITS, low SPECIFICITY, and high ENERGY. However, too low structural difference might cause high SPECIFICITY, enrichment factor (EF), and Güner-Henry score (GH), which would be likely detrimental for further screening. The model_6 was considered as the optimal pharmacophore model as it gave relative fitting parameters, including SPECIFICITY = 5.709 (>5), N_HITS ≈ 12, FEATS = 8, PARETO = 0, ENERGY = 9.41, STERICS = 1864.20, HBOND = 486.00, and MOL_QRY = 104.79 [27]. Additionally, the calculated parameters of a decoy set method for the model_6 could be concluded as follows: GH = 0.75 (0.6 < GH < 0.8) and EF = 120.56 (> 1). These also indicated that the model_6 has powerful ability to differentiate benign from inert compounds [28]. Therefore, the model_6 was chosen to analyze the key pharmacological features and was applied for the following virtual screening.

**Figure 7:**
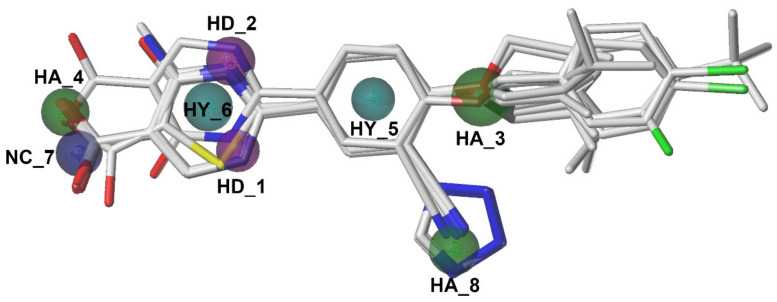
The optimal pharmacophore model. Green, magenta, cyan, and blue spheres represent hydrogen-bond acceptor atoms (HAs), hydrogen-bond donor atoms (DAs), hydrophobes (HYs), and negative centers (NCs), respectively.

**Figure 8:**
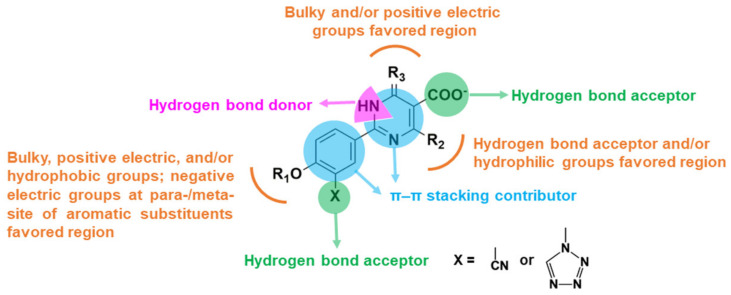
The SARs of 1,6-dihydropyrimidine-5-carboxylic acid XOIs based on the 3D-QSAR models, molecular docking results, and the pharmacophore model.

The pharmacophore features were shown in Figure 7 with the alignment of 12 XOIs. There were 3 green, 2 magenta, 2 cyan, and 1 blue spheres, which represented 3 hydrogen-bond acceptor atoms (HAs), 2 hydrogen-bond donor atoms (HDs), 2 hydrophobic centers (HYs), and 1 negative center (NC), respectively. It could be observed that the HDs covered the nitrogen atoms of the pyridine ring, suggesting that the nitrogen-containing heterocyclic structure might be indispensable for the activity. There were 2 HAs at the oxygen atoms of -OR_1_ and the carboxyl group, respectively, and another HA at the tetrazole or the cyano group, indicating that HA groups might be essential for activity in this position. The common scaffolds of febuxostat analogues were reported to be necessary to maintain the activity, which was consistent with the pharmacophore results that 2 HYs were located at the center of the 2 aromatic rings. These pharmacophore characteristics were generally in agreement with the 3D-QSAR and docking results. A graphical SAR summary of these ODC XOIs based on the results of the 3D-QSAR models, the molecular dockings, and the optimal pharmacophore model was shown in Figure 8.

### 2.5. Virtual Screening and Docking Analysis

The best pharmacophore model was converted into a UNITY query for the virtual screening from the ZINC purchasable database. The screened compounds (QFIT > 35) from the pharmacophore were then docked into the XO protein, and 37 compounds (docking score > 10) were obtained. We found that several screened compounds contained a similar indole ring that corresponded to the benzene ring of febuxostat analogues, which might provide new ideas for designing novel XOIs. The ADME properties of screened hits, compound **44**, and febuxostat were predicted by SwissADME (http://www.swissadme.ch (accessed on December 2019)), and a portion of data was summarized in Table 5 and Table S3. Finally, the screened hits **VS12** (ZINC71763654), **VS16** (ZINC09234838), **VS19** (ZINC59369278), and **VS26** (ZINC16688904) were obtained through the following criteria: 150 < molecular weight (MW) < 500 g/mol, 20 < topological polar surface area (TPSA) < 130 Å^2^, high gastrointestinal (GI) absorption, inexistent blood–brain barrier (BBB) permeability, synthetic accessibility (SA) score < 4.5, Lipinski violations = 0, and lower inhibitory characteristics with cytochrome P450 (CYP450) [21,29,30,31].

**Figure 9:**
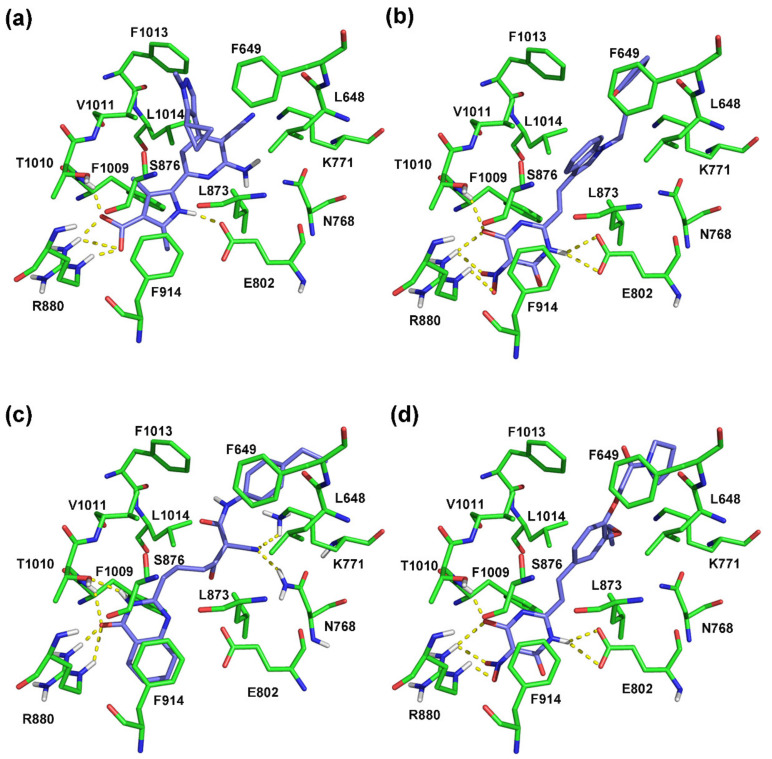
The docking results of the screened compounds **VS12** (**a**), **VS16** (**b**), **VS19** (**c**), and **VS26** (**d**) in the XO protein (PDB ID: 1N5X). The ligands, important residues, and hydrogen bonds were shown in blue sticks, green sticks, and yellow dashes, respectively.

The chemical structures and docking scores of compounds **VS12**, **VS16**, **VS19**, and **VS26** were summarized in Table 6, and their docking conformations were shown in Figure 9. Compounds **VS12**, **VS16**, and **VS26** could form similar hydrogen bonds with residues Arg880, Thr1010, and Glu802, which were similar with the docking results of febuxostat and compound **44**. In the previous docking results, residues Arg880, Thr1010, and Glu802 have also been proved to be essential for XOI binding. As for compound **VS19**, it could be observed to bind stably by hydrogen bonds with Arg880, Thr1010, Asn768, and Leu648. Compared with febuxostat and compound **44**, the nitrogen-containing heterocyclic rings of compounds **VS12**, **VS16**, **VS19**, and **VS26** were also observed to form π-π stacking interactions with Phe914 and Phe1009. These docking results demonstrated that the 4 screened compounds might have the potential to become novel XOIs, which might provide new ideas for designing novel XOI chemotypes.

### 2.6. Molecular Dynamics Simulations

**Figure 10:**
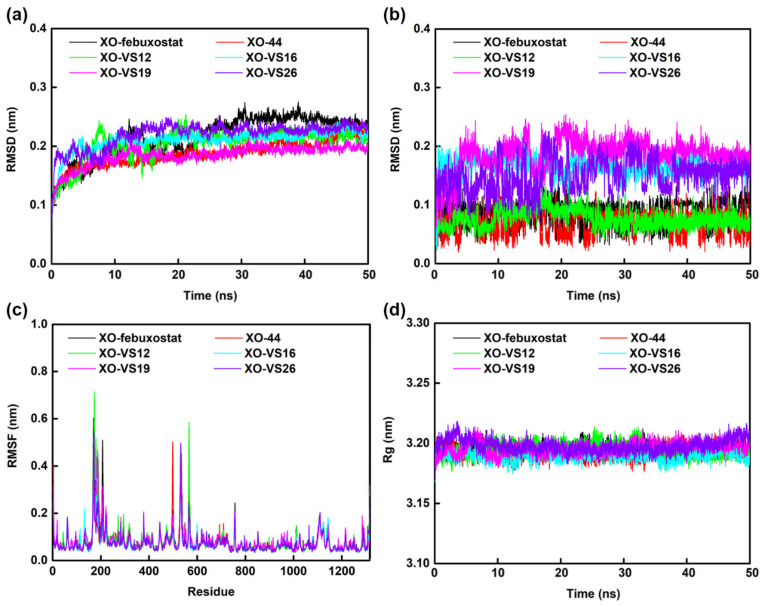
The results of the 50 ns MD simulations of complexes XO-febuxostat (black), XO-**44** (red), XO-**VS12** (green), XO-**VS16** (cyan), XO-**VS19** (magenta), and XO-**VS26** (violet). (**a**) The RMSDs of the XO backbone atoms. (**b**) The RMSDs of ligands (febuxostat, **44**, **VS12**, **VS16**, **VS19**, and **VS26**). (**c**) The residue RMSFs of XO. (**d**) The Rg of XO.

**Figure 11:**
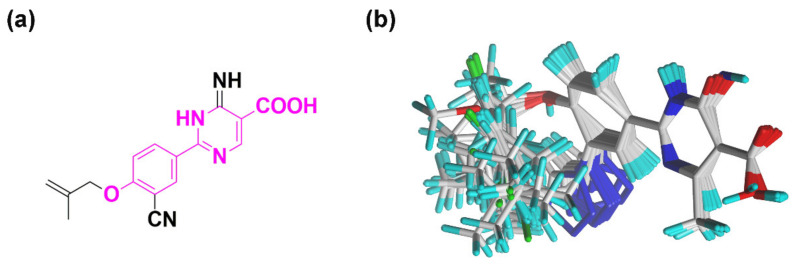
Molecular alignment for the 3D-QSAR models. (**a**) The common scaffold (magenta) used for the alignment. (**b**) The alignment result of all used XOIs.

The best docking conformations of compounds febuxostat, **44**, **VS12**, **VS16**, **VS19**, and **VS26** were subjected to MD simulations based on the repaired XO protein. To further analyze the stability of these protein–ligand complexes, the time-dependent behavior of complexes XO-**VS12**, XO-**VS16**, XO-**VS19**, and XO-**VS26** in the 50 ns simulation trajectories were analyzed using XO-febuxostat and XO-**44** as the comparisons, and their results were shown in Figure 10 and Figure 11, Figures S3–S5.

The RMSD values that could reflect the differences between the initial and current conformations were suited to evaluate the convergence and stability of the systems [32]. The comparisons between the initial and final conformations of 6 complexes were shown in Figure S3. According to Figure 10a, the RMSD values of backbone atoms in the 6 complexes could become stable at approximately 25 ns and varied around 0.2 nm. In complex XO-febuxostat, the RMSD values of backbone atoms were higher than those of the other complexes. As shown in Figure 10b, the ligands (febuxostat, **44**, **VS12**, **VS16**, **VS19**, and **VS26**) with low RMSD values could become stable during MD simulations. Compared to febuxostat, the RMSD results manifested that compounds **VS12**, **VS16** and **VS26** might be more favorable for binding to XO.

The root-mean-square fluctuation (RMSF) values (Figure 10c) were used to evaluate the flexibility of the protein side chains, and the RMSF values of >0.35 nm were considered as high flexibility [33]. The RMSF values of the most residues in the 6 systems showed similar fluctuation, and the critical residues in all complexes exhibited low flexibility, illustrating that the key interactions of all compounds in the binding pocket might similar. The gyration radius (Rg) values could estimate the protein compactness, and the numerical results of each system over time were summarized in Figure 10d. All complexes showed slight fluctuations, and the Rg values finally stabilized between 3.17 and 3.21 nm. The above evidence suggested that the protein conformations of all complexes were basically stable, which was consistent with the RMSD results [28].

The hydrogen-bond number were carried out to assess the stability of the complexes from another perspective [34]. Figure S4 showed the hydrogen-bond numbers between the ligand and protein in complexes XO-febuxostat, XO-**44**, XO-**VS12**, XO-**VS16**, XO-**VS19**, and XO-**VS26** fluctuated within 0–4, 0–6, 0–7, 0–4, 0–5, and 0–4, respectively, during the MD simulations. Furthermore, the hydrogen-bond numbers in complexes XO-febuxostat, XO-**44**, XO-**VS12**, XO-**VS16**, XO-**VS19**, and XO-**VS26** maintained in 2, 3, 3, 2, 1, and 1, respectively. The above results suggested that compound **VS12** might bind tightly with XO by more hydrogen bonds than the other virtual-screened compounds.

The binding free energy was calculated by the Molecular Mechanics-Poisson Bolzmann Surface Area (MM-PBSA) method, and the corresponding values are listed in Table 7. The binding free energies of compounds febuxostat, **44**, **VS12**, **VS16**, **VS19**, and **VS26** in the XO protein were −97.04, −95.30, −88.31, −159.71, −95.91, and −150.84 kJ/mol, respectively. These results indicated that the binding strengths of compounds **VS16** and **VS26** might be stronger than those of compounds febuxostat and **44** [35]. It was observed that binding energies of **VS16** and **VS26** with XO were more favorable due to their higher van der Waals interactions (ΔE_vdw_: **VS16** = −220.50 kJ/mol; **VS26** = −216.89 kJ/mol). In addition, **VS16** exhibited relatively low polar solvation energy (ΔG_PB_: 109.67 kJ/mol), and **VS26** showed relatively good electrostatic interaction (ΔE_ele_: −40.34 kJ/mol), respectively. As for all 6 systems, the ΔE_vdw_ played an important role in the ΔG_binding_, the contribution of ΔE_ele_ was nearly counteracted by the ΔG_PB_, and the values of SASA energy (ΔG_SA_) in each complex were semblable. These results indicated that the nonpolar energy might be the main driving force for the binding of these XOIs. To understand the stability of complexes, the binding energies obtained during the MD simulations versus time were shown in Figure S5. It was observed that the binding energies of all complexes reached stability after 45 ns. Essentially, the stability of compounds **VS12**, **VS16**, **VS19**, and **VS26** in the XO binding pocket were verified. The above analyses proved that the screened compounds might have powerful potential to be XOI hits.

## 3. Materials and Methods

### 3.1. Molecules Set and Optimization

All calculations and simulations were performed using SYBYL-X 2.1 software (Tripos Inc., St. Louis, MO, USA) running on Windows 7 workstations, unless otherwise specified. A dataset of 46 ODCs (Table 1) was selected from the published literature [3,4] for 3D-QSAR modelling. The energy and geometry minimizations of all ODC compounds were applied in the Tripos force field and the Gasteiger–Hückel charges under the energy gradient convergence criterion of 0.005 kcal/(mol·Å) and the maximum iteration coefficient of 10,000 [36].

### 3.2. Molecular Modeling and Molecular Alignment

The CoMFA and CoMSIA models were used to help us to better understand the relationship between the characteristics of molecular structures and their activities. SYBYL-X 2.1 software was used for the CoMFA and CoMSIA models. Different physicochemical properties used for the CoMFA and CoMSIA models are calculated by the same lattice boxes with the same sp^3^ carbon probe (default probe atom) [28]. Thereinto, the CoMFA model incorporates two different descriptor fields: steric and electrostatic fields. The steric and electrostatic fields were calculated using Lennard-Jones potential and Coulombic potential, respectively [37]. In CoMSIA, five different similarity fields, including steric, electrostatic, hydrophobic, HBD, and HBA fields, were calculated using the Gaussian function [38]. Besides, the CoMFA and CoMSIA models are transformed to contour maps using the field type of “StDev*Coeff”, which are useful for analyzing the SARs [6]. Forty-six compounds used for the 3D-QSAR models were randomly divided into a training set of 35 molecules for the model generation and a test set of 11 molecules for the model validation. The molecular alignment quality has a great effect on the predictability and robustness of the models [39]. In this study, the compound **44** with the highest potency was selected as a template, and the other molecules were superimposed onto the common skeleton (colored as magenta) by automatic alignment and manual adjustment. The alignment results of all ODCs were shown in Figure 11.

### 3.3. Model Validation

PLS regression analysis method was performed for establishing the 3D-QSAR models. The first step detecting the reproducibility and robustness of the CoMFA and CoMSIA models was the internal validation. In the PLS analysis, a series of parameters, including q^2^, ONC, R^2^, F, and SEE, were calculated to assess the predictive ability of models [36]. To further judge the feasibility of the constructed models, external validation parameters, including r_0_^2^, r_0_′^2^, k, k’, r_m_^2^, r’_m_^2^, r_pred_^2^, Δr_m_^2^, $\bar{r_{m}^{2}},$ and RMSE, were further taken into consideration [39]. r_0_^2^ (predicted vs. actual pIC_50_) and r_0_′^2^ (actual vs. predicted pIC_50_) are the correlation coefficients of regression lines with a zero intercept, and k (predicted vs. actual pIC_50_) and k’ (actual vs. predicted pIC_50_) are the slopes of regression lines, respectively. r_m_^2^, r_pred_^2^, and RMSE are calculated according to the following Equations (1)–(3), respectively.

(1) $$r_{m}{}^{2}=r^{2}\times \left(1-\sqrt{r^{2}-r_{0}{}^{2}}\right)\;$$

(2) $$r_{pred}{}^{2}=1-\frac{\sum {\left(y_{pred(test)}-y_{\left(test\right)}\right)}^{2}}{\sum {\left(y_{\left(test\right)}-\bar{y_{\left(training\right)}}\right)}^{2}}$$

(3) $$RMSE=\sqrt{\frac{1}{n}\sum {\left(y_{\left(test\right)}-y_{pred(test)}\right)}^{2}}$$

The y_(test)_, y_pred(test)_, and $\bar{y_{\left(\mathrm{training}\right)}}$ represent the actual pIC_50_ value of each test set compound, the predicted pIC_50_ value of each test set compound, and the mean pIC_50_ value of the training set compounds, respectively [40]. An appropriate model should satisfy the following conditions: q^2^ > 0.5, R^2^ > 0.8, $\frac{r^{2}-r_{0}^{2}\left({\mathrm{or}\;r}_{0}^{{}^{\prime }2}\right)}{r^{2}}$ < 0.1, 0.85 ≤ k (or k’) ≤ 1.15, Δr_m_^2^ < 0.2, $\bar{r_{m}^{2}}$ > 0.5, and r_pred_^2^ > 0.6 [41].

### 3.4. Molecular Docking

Molecular docking served as a helpful tool to obtain the reasonable binding conformations of bioactive molecules and to identify core residues in the active site of target protein. The crystal structure of bovine XO protein (PDB ID: 1N5X), a very close homologue of human XO enzyme, was used for molecular docking by the surflex-docking package of SYBYL-X 2.1 with default parameters [1]. The sequence alignment of bovine (*Bos taurus*) and human (*Homo sapiens*) XO with approximately 90% sequence identity was shown in Figure S6, and particularly in the febuxostat binding site, the key amino acids were the same, which was consistent with the reported literatures [1,20]. Before docking, an online web service (http://www.mrc-lmb.cam.ac.uk/pca/ (accessed on September 2020)) was used to explore the non-covalent contacts between ligand and protein [25]. After the pretreatment steps of the original protein, including hydrogenating, adding electric charges, extracting the crystallographic ligands, and removing water and other unnecessary atoms, the applicable docking pocket was generated with a threshold of 5 Å by a “ligand” mode. The crystallographic ligand was first redocked into the pocket to examine the dependability of the docking method. The conformation differences between the redocked and original ligands were evaluated by the RMSD values [17]. The RMSD < 2.0 Å is considered as a reference criterion, indicating that the docking method is reasonable. The same docking method was then applied on 46 ODC XOIs (Table 1), and the appropriate docking conformations with different docking scores were then obtained [42]. Finally, the conformations of febuxostat, the most active compound **44**, and the least active compound **26** were used for further analyses. The docking visualization was completed by PyMOL software (DeLano Scientific LLC, San Carlos, California, USA).

### 3.5. Pharmacophore Model

Pharmacophore model could be used to extract the key chemical information of active compounds, and to automatically generate pharmacophore features, for instance, HAs, HDs, HYs, and NCs [23]. Twelve molecules (Table 1) containing febuxostat with relatively high activities and diverse structures were selected to construct pharmacophore models by the Genetic Algorithm with Linear Assignment of Hypermolecular Alignment of Datasets (GALAHAD) module of SYBYL-X 2.1. Twenty models with different parameters were generated, of which the models with optimal SPECIFICITY, N_HITS, ENERGY, STERICS, HBOND, and MOL_QRY values were chosen for further studies. In addition, a decoy set method was used to assess the searching active molecules capability of pharmacophore models. The potential pharmacophore models were performed to screen a decoy set database. The decoy set database was composed of 6234 inactive compounds (downloaded from http://dud.docking.org/r2/ (accessed on September 2019)) and 35 active ODCs (Table 1), except for the 11 ODCs and febuxostat that used for constructing this model [28]. The EF and GH values used for evaluating the reliability of the models were calculated as follows:

(4) $$EF=\frac{H_{a}/H_{t}}{A/D}$$

(5) $$GH=\left[\frac{H_{a}(3A+Ht)}{4H_{t}A}\right]\left(1-\frac{H_{t}-H_{a}}{D-A}\right)$$

in which H_a_, H_t_, A, and D represent the number of true positive compounds in the hit list, the number of all compounds in the hit list, the number of true positive compounds in the database, and the number of all compounds in the database, respectively [43]. When EF > 1 and 0.6 < GH < 0.8, the model is eligible to be chosen for further analyses and virtual screening.

### 3.6. Virtual Screening

A multi-stage virtual screening was carried out against the purchasable ZINC15 database (http://zinc15.docking.org (accessed on September 2019)) [44] by the combination of the optimal pharmacophore model, molecular dockings, and ADME predictions. The pharmacophore features from the best pharmacophore model were extracted to use as a search query for the first-round screening. The QFIT parameters in the range of 0 to 100 were obtained to assess the matching degree of screened compounds with the pharmacophore features [33]. The compounds with a QFIT > 35 were then selected for the second-round screening of docking. The compounds with a docking score > 10 were selected for the third-round screening [13]. In the third-round screening, the ADME profiling was then applied to assess pharmacokinetic and pharmacodynamic properties of the second-round screened compounds by a web tool of SwissADME (http://www.swissadme.ch (accessed on December 2019)) [30]. Among the predicted ADME properties, following properties were considered preferentially to get the satisfied compounds, including MW, TPSA, GI absorption, BBB permeability, inhibitory ability assessment of CYP450, Lipinski violations, and SA score [10,29,30,31]. Finally, the hits compounds with desired pharmacophore compliance, preferable docking scores, and ideal ADME evaluation results were further investigated for their binding interactions and stability in the XO protein by MD simulations and post-analysis experiments.

### 3.7. Molecular Dynamics Simulations

To further verify the stability of the docked hits compounds in the XO protein, 50 ns MD simulations were performed on different complexes using GROMACS 2016.5 software (Uppsala University, Stockholm University, and the Royal Institute of Technology, Sweden). The overall XO protein was a homodimer, in which one of subunit was selected for simulations. Residue deficiencies in 166–191 and 532–536 (loop region) were repaired by the protein loop search module of SYBYL-X 2.1 software, and the residues 1326–1332 were reasonably removed to solve the problem of the residues 1317–1325 deficiencies in the subunit terminal. The repaired models with good fit and high homology were further evaluated using PROCHECK and ProSa servers. Compared with the evaluation results of the original and optimal repaired proteins (Figure S7), the residues in disallowed regions were unchanged, and the Z-score value showed little difference, indicating the repaired protein was eligible enough for simulations. The protein topology files were generated by the pdb2gmx program under the AMBER99SB force field [1]. The ligand topological files were obtained by the ACPYPE program [35]. The protein–ligand complexes were positioned into the center of a cubic box with a side length of 13.7 nm, and there was a buffering distance of approximately 12 Å between the protein periphery and the box edges. This box filled with water, and five additional chloride ions, were added into box to reach the neutralization. The steepest gradient descent method was then used to minimize the energy of each system within 1 ns (50,000 steps) with a convergence criterion of 10 kJ/mol [32]. The protein–ligand complex was subjected to 100 ps simulation to achieve the NVT and NPT equilibrium at 300 K and 1 atm, respectively [28]. Finally, 50 ns MD simulations were performed for all systems, and their trajectories were recorded at every 10 ps (5000 steps) for post-analysis [26]. Many post-analyses, such as RMSD, RMSF, Rg, and hydrogen-bond numbers, were conducted to investigate the stability or variation of all complexes during the dynamic environment. The equilibrium trajectory (the last 5 ns) of the MD simulations was extracted to calculate the binding free energy using the MM-PBSA method. The binding free energy was calculated as follows:

(6) $$\Delta G_{bind}=G_{complex}-G_{free-protein}-G_{free-ligand}$$

in which G_complex_, G_free-protein_, and G_free-ligand_ represent the free energy of protein–ligand complex, protein, and ligand, respectively [33].

## 4. Conclusions

In this work, an integrated computational study, including 3D-QSAR models, molecular dockings, pharmacophore models, and MD simulations, was performed on 46 novel ODC XOIs. 3D-QSAR models with good statistical parameters were constructed to provide significant insights into the SARs of these ODCs. Molecular docking results indicated that residues Glu802, Arg880, Thr1010, and Asn768 could form hydrogen bonds with these XOIs, and residues Phe914 and Phe1009 could also form π-π interactions with them. These interactions might be essential for their affinity with the XO protein. The best pharmacophore model with eight features was inconsistent with the 3D-QSAR and docking results. Four hit compounds (**VS12**, **VS16**, **VS19**, and **VS26**) with ideal compatibility for the pharmacophore model, relatively high docking scores, and good ADME characteristics were retrieved by the multi-round virtual screening. The MD simulations also indicated hits **VS12**, **VS16**, **VS19**, and **VS26** could bind well with the XO protein during the dynamic environment. We expect that these results will be helpful for the design and development of novel XOIs, and the screened compounds could provide more alternatives and ideas for XOIs.

## Figures and Tables

**Table 1:**
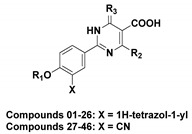
Chemical structures of the used non-purine XOIs and their actual and predicted pIC_50_ values.

No.	R_1_	R_2_	R_3_	IC_50_ (µM)	pIC_50_	CoMFA		CoMSIA	
						Predicted pIC_50_	Residuals	Predicted pIC_50_	Residuals
01 *	methyl	H	O	0.0920	7.0362	6.984	0.0522	7.075	0.0388
02	*iso*-propyl	H	O	0.0737	7.1325	7.167	0.0345	7.132	0.0005
03	*iso*-butyl	H	O	0.0644	7.1911	7.204	0.0129	7.192	0.0009
04	*iso*-pentyl	H	O	0.0541	7.2668	7.25	0.0168	7.255	0.0118
05 *^,#^	allyl	H	O	0.0437	7.3595	7.407	0.0475	7.329	0.0305
06	*iso*-butenyl	H	O	0.0569	7.2449	7.246	0.0011	7.253	0.0081
07	*iso*-pentenyl	H	O	0.0692	7.1599	7.163	0.0031	7.146	0.0139
08^#^	propinyl	H	O	0.0500	7.301	7.263	0.038	7.302	0.001
09^#^	methylene cyclopropane	H	O	0.0461	7.3363	7.324	0.0123	7.328	0.0083
10	cyclopentyl	H	O	0.0585	7.2328	7.275	0.0422	7.235	0.0022
11	methylene cyclohexane	H	O	0.0683	7.1656	7.143	0.0226	7.144	0.0216
12	benzyl	H	O	0.0945	7.0246	7.102	0.0774	7.018	0.0066
13	*p*-methylbenzyl	H	O	0.0894	7.0487	7.074	0.0253	7.106	0.0573
14 *	*p*-tert-butylbenzyl	H	O	0.1490	6.8268	6.804	0.0228	6.801	0.0258
15	*p*-methoxylbenzyl	H	O	0.0507	7.295	7.330	0.035	7.301	0.006
16	*p*-fluorobenzyl	H	O	0.0531	7.2749	7.226	0.0489	7.211	0.0639
17	*p*-chlorobenzyl	H	O	0.0691	7.1605	7.195	0.0345	7.201	0.0405
18	*p*-bromobenzyl	H	O	0.0552	7.2581	7.194	0.0641	7.233	0.0251
19	*m*-methoxylbenzyl	H	O	0.0516	7.2874	7.273	0.0144	7.301	0.0136
20	*m*-fluorobenzyl	H	O	0.0477	7.3215	7.298	0.0235	7.319	0.0025
21 *^,#^	*m*-chlorobenzyl	H	O	0.0288	7.5406	7.593	0.0524	7.535	0.0056
22	*m*-bromobenzyl	H	O	0.0450	7.3468	7.329	0.0178	7.342	0.0048
23	*o*-chlorobenzyl	H	O	0.0917	7.0376	7.062	0.0244	7.004	0.0336
24	2,5-dichlorobenzyl	H	O	0.0639	7.1945	7.120	0.0745	7.215	0.0205
25	2,4-dichlorobenzyl	H	O	0.0838	7.0768	7.129	0.0522	7.116	0.0392
26	hydrogen	H	O	0.6290	6.2013	6.205	0.0313	6.201	0.0003
27	*iso*-propyl	H	O	0.0916	7.0381	7.013	0.0222	7.042	0.0039
28 *	*iso*-butyl	H	O	0.0609	7.2154	7.238	0.0226	7.270	0.0546
29 *^,#^	*iso*-pentyl	H	O	0.0250	7.6021	7.561	0.0411	7.705	0.1029
30 *	allyl	H	O	0.0811	7.091	7.107	0.016	7.026	0.065
31 ^#^	*iso*-butenyl	H	O	0.0336	7.4737	7.505	0.0061	7.507	0.0333
32	*iso*-pentenyl	H	O	0.0388	7.4112	7.389	0.1031	7.429	0.0178
33 *	benzyl	H	O	0.0387	7.4123	7.297	0.1153	7.405	0.0073
34	*p*-fluorobenzyl	H	O	0.0382	7.4179	7.424	0.0998	7.398	0.0199
35	*p*-chlorobenzyl	H	O	0.0499	7.3019	7.405	0.06	7.410	0.1081
36 ^#^	*p*-bromobenzyl	H	O	0.0298	7.5258	7.426	0.0116	7.462	0.0638
37 *^,#^	*p*-tert-butylbenzyl	H	O	0.1970	6.7055	6.874	0.1685	6.787	0.0815
38	*p*-methylbenzyl	H	O	0.0354	7.451	7.391	0.06	7.380	0.071
39	*iso*-pentyl	CH_3_	O	0.5400	6.2676	6.256	0.0116	6.248	0.0196
40	*iso*-butenyl	CH_3_	O	0.5677	6.2459	6.253	0.0071	6.263	0.0171
41 *	*p*-bromobenzyl	CH_3_	O	0.1854	6.7319	6.822	0.0901	6.512	0.2199
42 *	*p*-methylbenzyl	CH_3_	O	0.1590	6.7986	6.807	0.0084	6.469	0.3296
43 ^#^	*iso*-pentyl	H	NH	0.0240	7.6198	7.664	0.0442	7.642	0.0222
44 ^#^	*iso*-butenyl	H	NH	0.0181	7.7423	7.684	0.0583	7.708	0.0343
45 ^#^	*p*-bromobenzyl	H	NH	0.0271	7.567	7.58	0.013	7.558	0.009
46	*p*-methylbenzyl	H	NH	0.0339	7.4698	7.528	0.0582	7.487	0.0172
Febuxostat ^#^	-	-	-	0.0236	7.6271	-	-	-	-

* The test set compounds used for the 3D-QSAR models. ^#^ The compounds used for the pharmacophore models.

**Table 2:** Internal statistical parameters of the CoMFA and CoMSIA models.

	Model	q^2^	ONC	SEE	R^2^	F	r_pred_^2^	Field Contribution (%)				
								S	E	H	D	A
CoMFA	S + E	0.897	7	0.050	0.983	229.50	0.948	0.773	0.227			
CoMSIA	S + E + H + D + A	0.922	11	0.041	0.990	212.26	0.840	0.105	0.248	0.372	0.193	0.082
	H + D	0.907	12	0.048	0.987	141.51	0.670			0.763	0.237	
	S + E + D	0.931	7	0.055	0.980	189.71	0.665	0.175	0.404		0.421	
	E + H + A	0.875	11	0.043	0.989	195.52	0.871		0.331	0.528		0.141
	S + E + H + D	0.926	10	0.041	0.990	232.65	0.825	0.107	0.257	0.422	0.214	
	E + H + D + A	0.921	11	0.042	0.990	205.711	0.848		0.271	0.436	0.204	0.089

q^2^: cross-validated correlation coefficient; ONC: optimal number of components; SEE: standard error of estimate; R^2^: non-cross-validated correlation coefficient; F: F-statistic values; r_pred_^2^: predictive correlation coefficient; S: steric fields; E: electrostatic fields; H: hydrophobic fields; D: hydrogen bond donor fields; A: hydrogen bond acceptor fields.

**Table 3:** External validation parameters of the CoMFA and CoMSIA models.

Validation Parameters	RMSE	r^2^	r_0_^2^	r’_0_^2^	(r^2^ − r’_0_^2^)/r^2^	k	k’	r_m_^2^	r’_m_^2^	Δr_m_^2^	$\bar{r_{m}^{2}}$
CoMFA	0.074	0.950	0.946	0.936	0.0147	0.998	1.002	0.890	0.838	0.052	0.864
CoMSIA (S + E + H + D + A)	0.130	0.922	0.840	0.897	0.027	1.005	0.995	0.658	0.776	0.118	0.717

RMSE: root mean square error for the test set compounds; r^2^: the regression line coefficient of correlation for the test set compounds; r_0_^2^ (predicted vs. observed activities) and r_0_′^2^ (observed vs. predicted activities): the correlation coefficient of regression lines with a zero intercept; k (predicted vs. observed activities) and k′ (observed vs. predicted activities): the slope of regression lines with a zero intercept; r_m_^2^: calculated by [r^2^ (1−(r^2^ − r_0_^2^)^0.5^)]; r’_m_^2^: calculated by [r^2^ (1−(r^2^ − r_0_′^2^)^0.5^)]; Δr_m_^2^ and $\bar{r_{m}^{2}}$: the difference and average values between r_m_^2^ and r_m_′^2^.

**Table 4:** Statistical results of the pharmacophore models.

Name	SPECIFICITY	N_HITS	FEATS	PARETO	ENERGY	STERICS	HBOND	MOL_QRY
Model_1	4.629	11	8	0	8.76	2052.10	497.60	84.69
Model_2	4.630	11	8	0	9.53	2064.40	498.40	81.58
Model_3	2.330	9	10	0	8.47	2023.00	499.90	65.32
Model_4	4.651	11	8	0	11.39	1986.30	494.50	87.28
Model_5	4.629	11	8	0	10.10	1911.90	492.00	91.15
Model_6	5.709	11	8	0	9.41	1864.20	486.00	104.79
Model_7	3.440	12	10	0	11.68	1834.50	495.80	117.25
Model_8	4.630	11	8	0	10.80	1840.80	503.10	85.90
Model_9	3.440	12	10	0	12.21	1835.30	494.70	117.25
Model_10	4.629	11	8	0	22.58	2149.70	496.10	85.45
Model_11	4.628	11	8	0	10.47	1965.60	490.20	85.45
Model_12	4.649	11	8	0	12.18	1996.40	487.40	93.3
Model_13	4.630	11	8	0	1240.81	2059.40	497.30	85.90
Model_14	4.630	11	8	0	10.80	1786.20	499.50	85.90
Model_15	3.440	12	10	0	9.89	1780.70	484.10	141.11
Model_16	3.440	12	10	0	14.36	1750.50	492.50	152.73
Model_17	3.439	12	10	0	10.26	1929.00	470.00	129.80
Model_18	4.637	11	8	0	10.82	1830.30	487.90	97.00
Model_19	3.441	12	10	0	10.19	1723.60	485.60	130.83
Model_20	4.636	11	8	0	1385.32	2189.60	481.20	97.00

**Table 5:** The ADME prediction results of the virtual-screened hits (**VS12**, **VS16**, **VS19**, and **VS26**), compound **44**, and febuxostat.

Parameter	Compound					
	VS12	VS16	VS19	VS26	Febuxostat	44
MW (g/mol)	375.4	417.39	401.44	429.4	316.37	310.31
Fraction Csp^3^	0.3	0.09	0.22	0.35	0.31	0.12
Rotatable bonds	4	7	8	9	5	5
TPSA (Å^2^)	113.4	105.7	98.6	130.3	111.5	122.9
GI absorption	High	High	High	High	High	High
BBB permeant	No	No	No	No	No	No
CYP1A2 inhibitor	Yes	No	Yes	No	Yes	No
CYP2C19 inhibitor	No	Yes	No	Yes	Yes	No
CYP2C9 inhibitor	No	No	No	No	Yes	No
CYP2D6 inhibitor	No	No	No	No	No	No
CYP3A4 inhibitor	No	No	No	No	No	No
Lipinski violations	0	0	0	0	0	0
SA score	3.4	3.32	3.25	3.34	3.12	2.64

**Table 6:** Chemical structures and docking scores of the virtual-screened hits obtained by the third-round screening.

Hit Compound	VS12	VS16	VS19	VS26
Structure	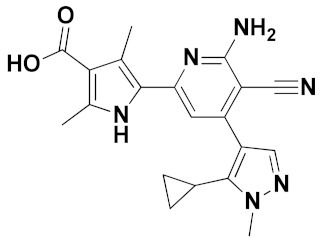	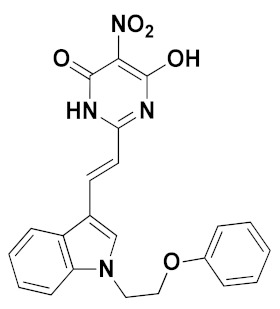	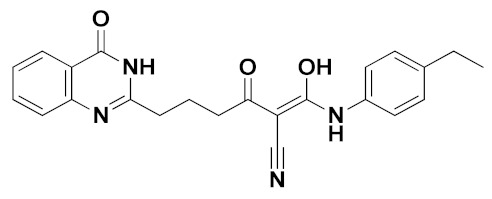	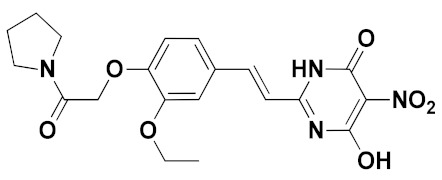
Docking score	10.62	11.87	10.76	11.03

**Table 7:** The free binding energies (kJ/mol) of febuxostat, compound **44**, and the virtual-screened hits (**VS12**, **VS16**, **VS19**, and **VS26**) in the XO protein.

Complex	ΔE_vdW_	ΔE_ele_	ΔG_PB_	ΔG_SA_	ΔG_binding_
XO-febuxostat	−175.32 ± 8.81	−39.95 ± 6.37	136.43 ± 11.05	−18.20 ± 0.73	−97.04 ± 9.45
XO-**44**	−160.03 ± 10.29	−107.25 ± 10.15	189.25 ± 11.17	−17.27 ± 0.78	−95.30 ± 8.32
XO-**VS12**	−195.31 ± 9.21	−69.99 ± 13.91	197.26 ± 13.45	−20.27 ± 0.81	−88.31 ± 10.40
XO-**VS16**	−220.50 ± 9.93	−28.10 ± 8.40	109.67 ± 10.71	−20.78 ± 0.86	−159.71 ± 11.41
XO-**VS19**	−152.19 ± 8.97	−49.01 ± 6.76	124.32 ± 11.20	−19.03 ± 0.86	−95.91 ± 10.52
XO-**VS26**	−216.89 ± 9.57	−40.34 ± 8.46	127.33 ± 14.15	−20.94 ± 0.91	−150.84 ± 9.61

## Data Availability

Data is contained within the article and Supplementary Materials.

## Supplementary Materials

Supplementary materials are available online at https://www.mdpi.com/article/10.3390/ijms22158122/s1.
