# Supplementary material for: Exploration of Novel Xanthine Oxidase Inhibitors Based on 1,6-Dihydropyrimidine-5-Carboxylic Acids by an Integrated in Silico Study
Source: Int J Mol Sci. 2021 Jul 29;22(15):8122. doi: 10.3390/ijms22158122 (PMC8348919; doi:10.3390/ijms22158122)
Supplement: Supplementary file 1 [file ijms-22-08122-s001.zip › ijms-1259737-supplementary.pdf]

Supporting Information

Exploration of Novel Xanthine Oxidase Inhibitors Based on 1,6-Dihydropyrimidine-5-Carboxylic Acids by an Integrated in Silico Study

Na Zhai [1], Chenchen Wang [1], Fengshou Wu [1], Liwei Xiong [1,2,*], Xiaogang Luo [1,3],

Xiulian Ju [1], Genyan Liu [1,*]

[a]*Hubei Key Laboratory of Novel Reactor and Green Chemical Technology, School of Chemical Engineering and Pharmacy, Wuhan Institute of Technology, Wuhan 430205, China*

[b] *Hubei Key Laboratory of Plasma Chemistry and Advanced Materials, Wuhan Institute of Technology, Wuhan 430205, China*

[c] *School of Materials Science and Engineering, Zhengzhou University, No.100 Science Avenue, Zhengzhou 450001, China*

[*] *Correspondence: zhily2000@126.com (L.X.); liugenyan@yahoo.com (G.L.)*

Table S1. Chemical structures of the used non-purine XOIs with their actual pIC_{50} values and docking scores.

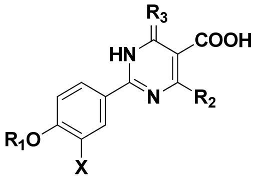

Compounds 01-26: X = 1H-tetrazol-1-yl
Compounds 27-46: X = CN

No.	R_1	R_2	R_3	IC_{50} (μM)	pIC_{50}	Docking score
01	methyl	H	O	0.0920	7.0362	8.98
02	*iso*-propyl	H	O	0.0737	7.1325	8.94
03	*iso*-butyl	H	O	0.0644	7.1911	9.44
04	*iso*-pentyl	H	O	0.0541	7.2668	9.38
05	allyl	H	O	0.0437	7.3595	9.55
06	*iso*-butenyl	H	O	0.0569	7.2449	9.46
07	*iso*-pentenyl	H	O	0.0692	7.1599	9.21
08	propinyl	H	O	0.0500	7.301	9.47
09	methylene cyclopropane	H	O	0.0461	7.3363	9.73
10	cyclopentyl	H	O	0.0585	7.2328	9.51
11	methylene cyclohexane	H	O	0.0683	7.1656	9.46
12	benzyl	H	O	0.0945	7.0246	8.97
13	*p*-methylbenzyl	H	O	0.0894	7.0487	9.38
14	*p*-tert-butylbenzyl	H	O	0.1490	6.8268	9.12
15	*p*-methoxylbenzyl	H	O	0.0507	7.295	9.61
16	*p*-fluorobenzyl	H	O	0.0531	7.2749	9.53
17	*p*-chlorobenzyl	H	O	0.0691	7.1605	9.35
18	*p*-bromobenzyl	H	O	0.0552	7.2581	9.46
19	*m*-methoxylbenzyl	H	O	0.0516	7.2874	9.46
20	*m*-fluorobenzyl	H	O	0.0477	7.3215	9.76
21	*m*-chlorobenzyl	H	O	0.0288	7.5406	10.63
22	*m*-bromobenzyl	H	O	0.0450	7.3468	9.67
23	*o*-chlorobenzyl	H	O	0.0917	7.0376	9.10
24	2,5-dichlorobenzyl	H	O	0.0639	7.1945	9.34
25	2,4-dichlorobenzyl	H	O	0.0838	7.0768	9.37
26	hydrogen	H	O	0.6290	6.2013	7.85
27	*iso*-propyl	H	O	0.0916	7.0381	9.02
28	*iso*-butyl	H	O	0.0609	7.2154	9.55
29	*iso*-pentyl	H	O	0.0250	7.6021	10.51
30	allyl	H	O	0.0811	7.091	9.22
31	*iso*-butenyl	H	O	0.0336	7.4737	9.91
32	*iso*-pentenyl	H	O	0.0388	7.4112	9.68
33	benzyl	H	O	0.0387	7.4123	9.78
34	*p*-fluorobenzyl	H	O	0.0382	7.4179	9.75
35	*p*-chlorobenzyl	H	O	0.0499	7.3019	9.58
36	*p*-bromobenzyl	H	O	0.0298	7.5258	10.51
37	*p*-tert-butylbenzyl	H	O	0.1970	6.7055	8.35
38	*p*-methylbenzyl	H	O	0.0354	7.451	9.99

39	*iso*-pentyl	CH_3	O	0.5400	6.2676	8.40
40	*iso*-butenyl	CH_3	O	0.5677	6.2459	8.36
41	*p*-bromobenzyl	CH_3	O	0.1854	6.7319	9.01
42	*p*-methylbenzyl	CH_3	O	0.1590	6.7986	8.81
43	*iso*-pentyl	H	NH	0.0240	7.6198	10.68
44	*iso*-butenyl	H	NH	0.0181	7.7423	10.69
45	*p*-bromobenzyl	H	NH	0.0271	7.567	10.50
46	*p*-methylbenzyl	H	NH	0.0339	7.4698	9.97

Table S2. The statistical results of other pharmacophore models using different compounds.

No.	SPECIFICITY	N_HITS	FEATS	PARETO	ENERGY	STERICS	HBOND	MOL_QRY
1	4.811	11	8	0	10.95	2156.90	562.20	95.77
2	4.803	12	8	0	11.80	2163.20	558.40	162.16
3	4.812	12	8	0	10.66	2143.50	562.30	87.70
4	5.638	11	8	0	10.54	2065.30	558.50	80.09
5	4.829	10	8	0	10.12	2191.80	561.10	95.43
6	4.808	12	8	0	8.20	2003.90	526.50	84.06
7	4.825	10	8	0	9.57	1847.80	548.20	76.72
8	4.824	11	8	0	11.54	2059.70	571.10	155.18
9	4.821	12	8	0	10.69	2267.10	584.60	78.31
10	4.822	11	8	0	24.33	2357.60	588.20	80.63

Table S3. The ADME prediction results of the non-ideal virtual-screened hits.

Hit compound	MW (g/mol)	Fraction Csp^3	Rotatable bonds	TPSA ($Å^2$)	GI absorption	BBB permeant	CYP1A2 inhibitor	CYP2C19 inhibitor	CYP2C9 inhibitor	CYP2D6 inhibitor	CYP3A4 inhibitor	Lipinski violations	SA score
VS01 (ZINC09434063)	422.61	0.6	13	142.1	Low	No	No	Yes	Yes	Yes	Yes	0	3.43
VS02 (ZINC04705438)	421.52	0.27	7	111	High	No	No	Yes	Yes	No	Yes	0	3.97
VS03 (ZINC59016051)	429.47	0.2	9	126.8	Low	No	No	Yes	Yes	No	Yes	0	3.62
VS04 (ZINC63625083)	427.5	0.23	6	103.1	High	No	No	No	No	No	No	0	4.41
VS05 (ZINC89942644)	318.37	0.38	7	103.2	High	No	No	No	No	No	No	0	3.26
VS06 (ZINC89942901)	370.43	0.44	8	149	Low	No	No	No	No	No	No	0	4.02
VS07 (ZINC04927833)	411.52	0.27	11	101.5	High	No	Yes	Yes	Yes	Yes	Yes	0	3.51
VS08 (ZINC95498920)	347.43	0.63	6	50.8	High	Yes	No	No	No	No	No	0	3.75
VS09 (ZINC39932293)	412.49	0.58	10	176.7	Low	No	No	No	No	No	Yes	1	4.7
VS10 (ZINC12537662)	469.45	0.05	9	151.1	Low	No	No	Yes	No	No	No	1	3.6
VS11 (ZINC12741060)	480.51	0.38	11	123.9	High	No	No	No	Yes	Yes	No	0	4.95
VS13 (ZINC24928355)	295.34	0.33	7	127.2	High	No	Yes	No	No	No	Yes	0	2.76
VS14 (ZINC06053573)	389.36	0.28	9	139.6	High	No	Yes	No	Yes	No	No	0	4.22
VS15 (ZINC31176221)	402.45	0.32	5	96.8	High	No	No	Yes	Yes	No	Yes	0	3.72
VS17 (ZINC21991916)	332.33	0.75	12	151.6	Low	No	No	No	No	No	No	0	3.61
VS18 (ZINC09493835)	463.51	0.47	10	191.3	Low	No	No	Yes	No	No	Yes	0	3.73
VS20 (ZINC65506066)	308.33	0.11	5	57.9	High	Yes	No	No	No	Yes	No	0	2.6
VS21 (ZINC09235475)	426.4	0.09	5	120.3	High	No	No	Yes	No	No	No	0	3.46
VS22 (ZINC32785893)	582.65	0.27	12	123.9	High	No	No	No	No	No	No	0	5.15
VS23 (ZINC21580816)	418.4	0.09	8	122.1	High	No	Yes	Yes	Yes	Yes	Yes	0	2.97
VS24 (ZINC63505952)	498.61	0.33	8	71.5	High	No	No	No	No	Yes	Yes	0	5.42
VS25 (ZINC09295542)	453.81	0.1	7	133.8	Low	No	No	Yes	No	No	No	0	3.47
VS27 (ZINC12820565)	384.44	0.19	6	120.4	High	No	Yes	Yes	Yes	Yes	Yes	0	3.17
VS28 (ZINC16362213)	348.42	0.35	9	106.6	High	No	Yes	Yes	Yes	No	Yes	0	2.87
VS29 (ZINC09120119)	455.55	0.27	8	158.5	Low	No	Yes	Yes	Yes	No	Yes	0	4.11
VS30 (ZINC72320942)	432.56	0.42	8	79.9	High	No	No	Yes	Yes	Yes	Yes	0	4.42

VS31 (ZINC12547951)	387.48	0.26	9	111.1	High	No	Yes	Yes	Yes	No	Yes	0	3.46
VS32 (ZINC39289590)	248.3	0.5	3	38.3	High	Yes	No	No	No	No	No	0	2.68
VS33 (ZINC73707897)	398.48	0.41	10	71.6	High	No	No	No	No	No	Yes	0	4.03
VS34 (ZINC18190685)	481.52	0.3	12	186.9	Low	No	No	No	No	No	Yes	0	3.84
VS35 (ZINC89942526)	369.44	0.37	9	128.2	High	No	No	No	No	No	No	0	3.54
VS36 (ZINC89943165)	370.47	0.5	8	101.4	High	No	No	No	No	No	No	0	3.77
VS37 (ZINC31167456)	502.92	0.43	11	173.7	Low	No	No	No	No	No	No	1	5.28

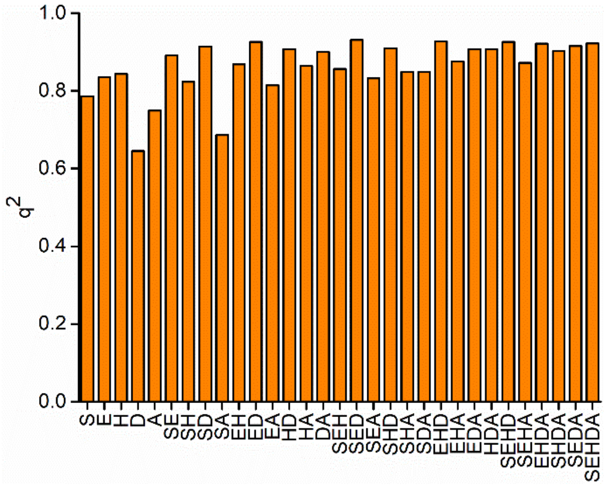

Figure S1. The q^2 values of all possible CoMSIA models with different field combinations (S: steric fields; E: electrostatic fields; H: hydrophobic fields; D: hydrogen-bond donor fields; A: hydrogen-bond acceptor fields).

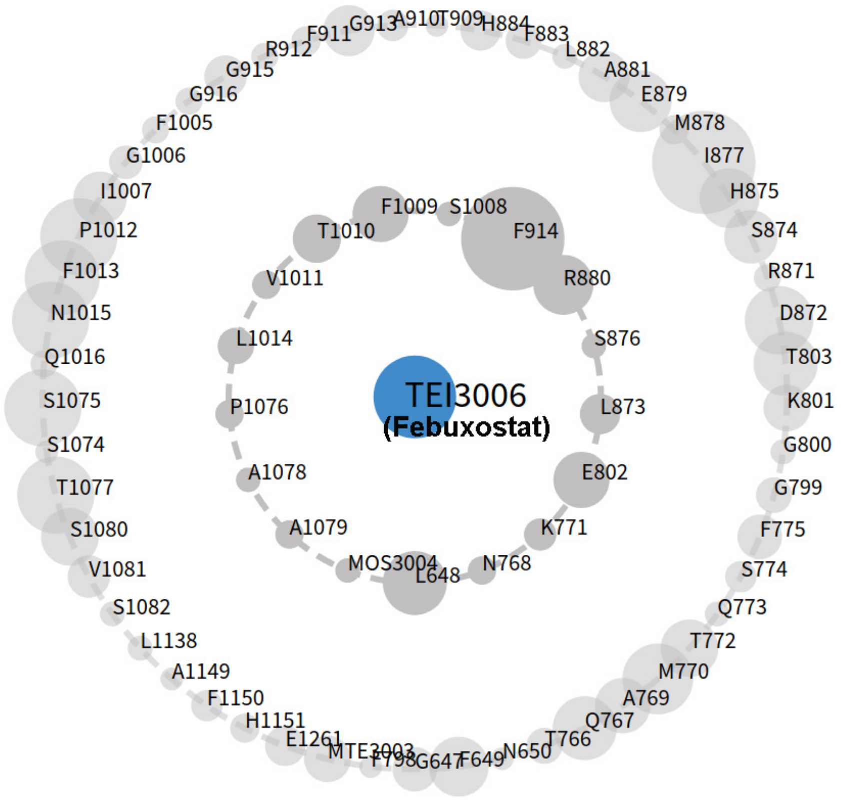

Figure S2. The asteroid plot of co-crystal XO structure (PDB ID: 1N5X) with febuxostat (center bule node).

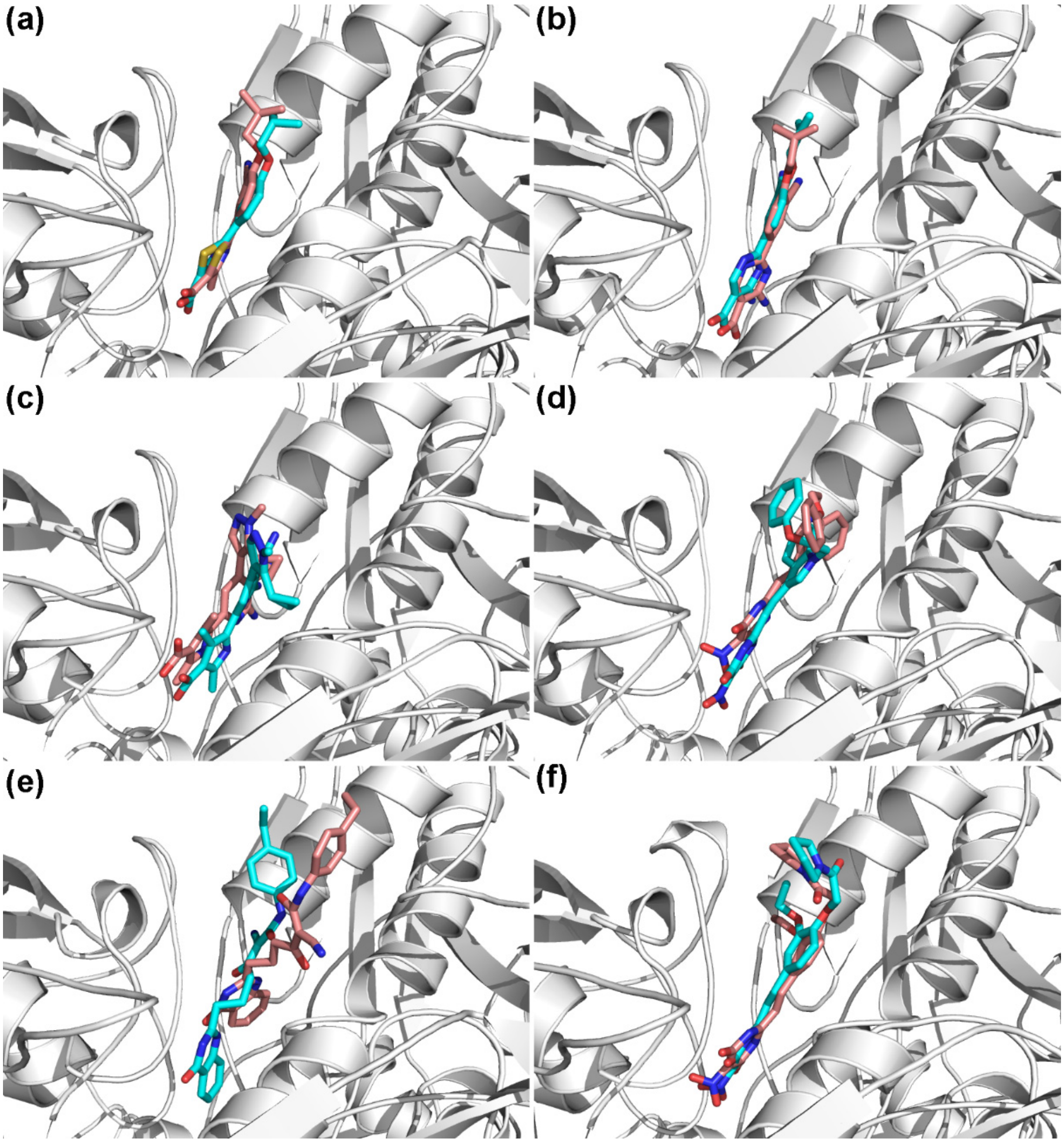

Figure S3. The initial (cyan sticks) and final (pink sticks) conformations of compounds febuxostat (a), **44** (b), **VS12** (c), **VS16** (d), **VS19** (e), and **VS26** (f) in XO protein during 50 ns MD simulations.

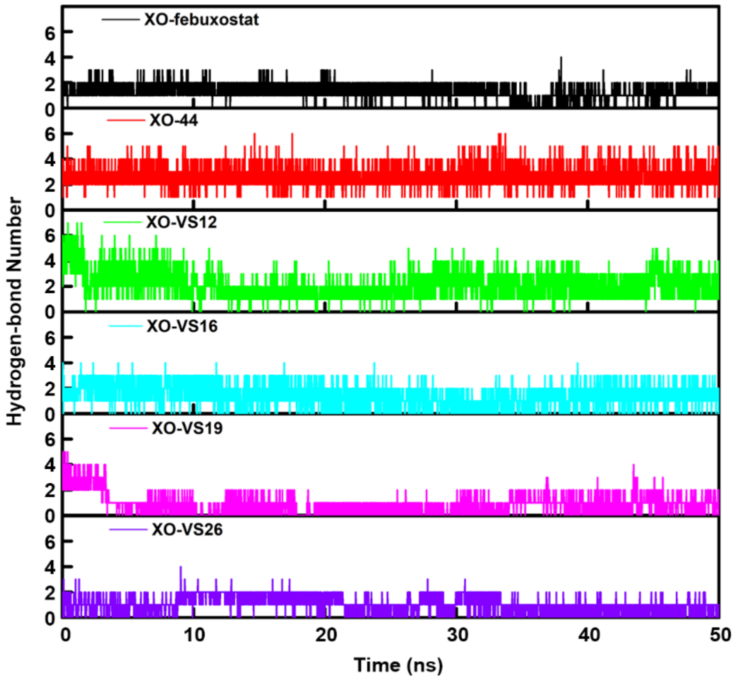

Figure S4. The hydrogen-bond numbers between protein and compounds febuxostat (black), **44** (red), **VS12** (green), **VS16** (cyan), **VS19** (magenta), and **VS26** (violet) during 50 ns MD simulations.

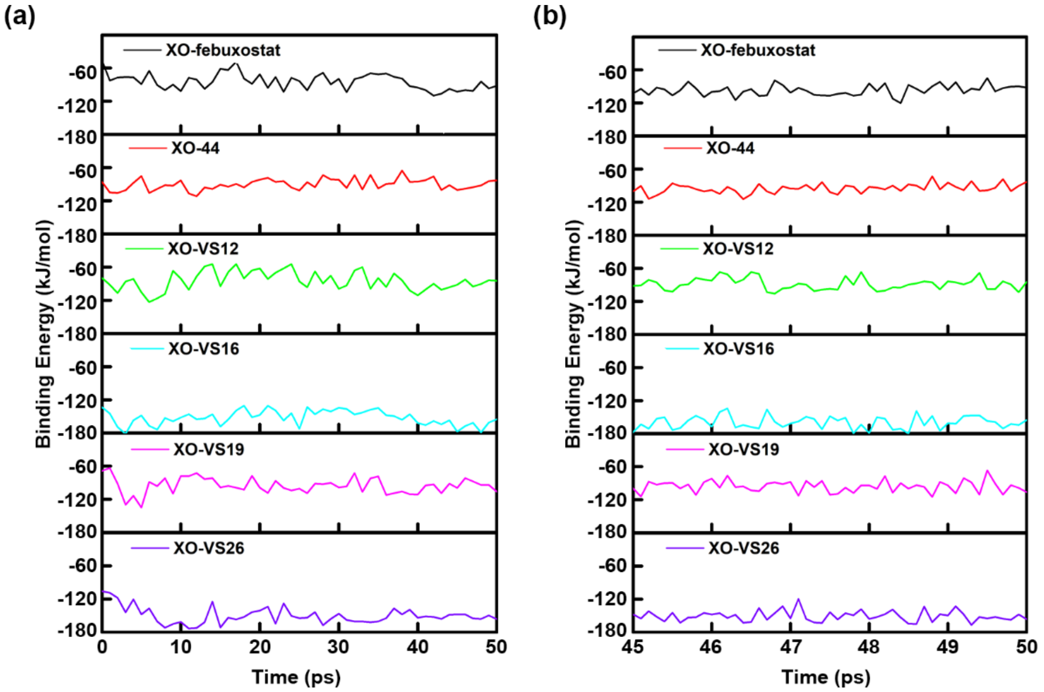

Figure S5. Binding energies (kJ/mol) convergence of complexes XO-febuxostat (black), XO-**44** (red), XO-**VS12** (green), XO-**VS16** (cyan), XO-**VS19** (magenta), and XO-**VS26** (violet) during 50 ns MD simulations (a: the 50 ns trajectory of each complex at an interval of 1 ns; b: the final 5 ns trajectory of each complex at an interval of 100 ps).

```
            1        10        20        30        40        50        60
XO_bovine   MTADELVFFVNGKKVVEKNADPETTLLAYLRRKLGLRGTKLGCGEGGCGACTVMLSKYDRLQDKIIH
XO_HUMAN    MTADKLVFFVNGRKVVEKNADPETTLLAYLRRKLGLSGTKLGCGEGGCGACTVMLSKYDRLQNKIVH

             70        80        90       100       110       120       130
XO_bovine   FSANACLAPICTLHHVAVTTVEGIGSTKTRLHPVQERIAKSHGSQCGFCTPGIVMSMYTLLRNQPEP
XO_HUMAN    FSANACLAPICSLHHVAVTTVEGIGSTKTRLHPVQERIAKSHGSQCGFCTPGIVMSMYTLLRNQPEP

               140       150       160       170       180       190       200
XO_bovine   TVEEIEDAFQGNLCRCTGYRPILQGFRTFAKNGGCCGGNGNNPNCCMNQKKDHTVTLSPSLFNPEEF
XO_HUMAN    TMEEIENAFQGNLCRCTGYRPILQGFRTFARDGGCCGGDGNNPNCCMNQKKDHSVSLSPSLFKPEEF

                  210       220       230       240       250       260
XO_bovine   MPLDPTQEPIFPPELLRLKDVPPKQLRFEGERVTWIQASTLKELLDLKAQHPEAKLVVGNTEIGIEM
XO_HUMAN    TPLDPTQEPIFPPELLRLKDTPRKQLRFEGERVTWIQASTLKELLDLKAQHPDAKLVVGNTEIGIEM

           270       280       290       300       310       320       330
XO_bovine   KFKNQLFPMIICPAWIPELNAVEHGPEGISFGAACALSSVEKTLLEAVAKLPTQKTEVFRGVLEQLR
XO_HUMAN    KFKNMLFPMIVCPAWIPELNSVEHGPDGISFGAACPLSIVEKTLVDAVAKLPAQKTEVFRGVLEQLR

               340       350       360       370       380       390       400
XO_bovine   WFAGKQVKSVASLGGNIITASPISDLNPVFMASGTKLTIVSRGTRRTVPMDHTFFPSYRKTLLGPEE
XO_HUMAN    WFAGKQVKSVASVGGNIITASPISDLNPVFMASGAKLTLVSRGTRRTVQMDHTFFPGYRKTLLSPEE

                  410       420       430       440       450       460
XO_bovine   ILLSIEIPYSREDEFFSAFKQASRREDDIAKVTCGMRVLFQPGSMQVKELALCYGGMADRTISALKT
XO_HUMAN    ILLSIEIPYSREGEYFSAFKQASRREDDIAKVTSGMRVLFKPGTTEVQELALCYGGMANRTISALKT

          470       480       490       500       510       520       530
XO_bovine   TQKQLSKFWNEKLLQDVCAGLAEELSLSPDAPGGMIEFRRTLTLSFFFKFYLTVLKKLGKD.SKDKC
XO_HUMAN    TQRQLSKLWKEELLQDVCAGLAEELHLPPDAPGGMVDFRCTLTLSFFFKFYLTVLQKLGQENLEDKC

               540       550       560       570       580       590       600
XO_bovine   GKLDPTYTSATLLFQKHPPANIQLFQEVPNGQSKEDTVGRPLPHLAAAMQASGEAVYCDDIPRYENE
XO_HUMAN    GKLDPTFASATLLFQKDPPADVQLFQEVPKGQSEEDMVGRPLPHLAADMQASGEAVYCDDIPRYENE

                  610       620       630       640       650       660
XO_bovine   LFLRLVTSTRAHAKIKSIDVSEAQKVPGFVCFLSADDIPGSNETGLFNDETVFAKDTVTCVGHIIGA
XO_HUMAN    LSLRLVTSTRAHAKIKSIDTSEAKKVPGFVCFISADDVPGSNITGICNDETVFAKDKVTCVGHIIGA

          670       680       690       700       710       720       730
XO_bovine   VVADTPEHAERAAHVVKVTYEDLPAIITIEDAIKNNSFYGSELKIEKGDLKKGFSEADNVVSGELYI
XO_HUMAN    VVADTPEHTQRAAQGVKITYEELPAIITIEDAIKNNSFYGPELKIEKGDLKKGFSEADNVVSGEIYI

             740       750       760       770       780       790       800
XO_bovine   GGQDHFYLETHCTIAIPKGEEGEMELFVSTQNAMKTQSFVAKMLGVPVNRILVRVKRMGGGFGGKET
XO_HUMAN    GGQEHFYLETHCTIAVPKGEAGEMELFVSTQNTMKTQSFVAKMLGVPANRIVVRVKRMGGGFGGKET

                810       820       830       840       850       860       870
XO_bovine   RSTLVSVAVALAAYKTGHPVRCMLDRNEDMLITGGRHPFLARYKVGFMKTGTIVALEVDHYSNAGNS
XO_HUMAN    RSTVVSTAVALAAYKTGRPVRCMLDRDEDMLITGGRHPFLARYKVGFMKTGTVVALEVDHFSNVGNT

                   880       890       900       910       920       930
XO_bovine   RDLSHSIMERALFHMDNCYKIPNIRGTGRLCKTNLSSNTAFRGFGGPQALFIAENWMSEVAVTCGLP
XO_HUMAN    QDLSQSIMERALFHMDNCYKIPNIRGTGRLCKTNLPSNTAFRGFGGPQGMLIAECWMSEVAVTCGMP

             940       950       960       970       980       990      1000
XO_bovine   AEEVRWKNMYKEGDLTHFNQRLEGFSVPRCWDECLKSSQYYARKSEVDKFNKENCWKKRGLCIIPTK
XO_HUMAN    AEEVRRKNLYKEGDLTHFNQKLEGFTLPRCWEECLASSQYHARKSEVDKFNKENCWKKRGLCIIPTK

              1010      1020      1030      1040      1050      1060      1070
XO_bovine   FGISFTVPFLNQAGALIHVYTDGSVLVSHGGTEMGQGLHTKMVQVASKALKIPISKIYISETSTNTV
XO_HUMAN    FGISFTVPFLNQAGALLHVYTDGSVLLTHGGTEMGQGLHTKMVQVASRALKIPTSKIYISETSTNTV

                 1080      1090      1100      1110      1120      1130
XO_bovine   PNSSPTAASVSTDIYGQAVYEACQTILKRLEPFKKKNPDGSWEDWVMAAYQDRVSLSTTGFYRTPNL
XO_HUMAN    PNTSPTAASVSADLNGQAVYAACQTILKRLEPYKKKNPSGSWEDWVTAAYMDTVSLSATGFYRTPNL
```

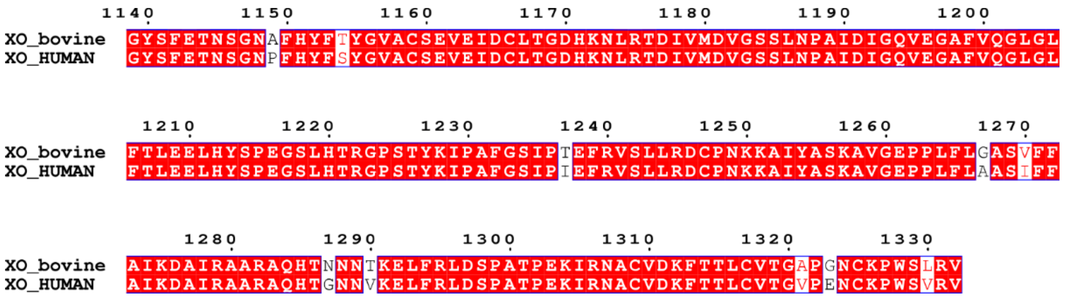

Figure S6. The sequence alignment of bovine and human XO enzymes (the key residues for febuxostat binding with XO marked by black frames).

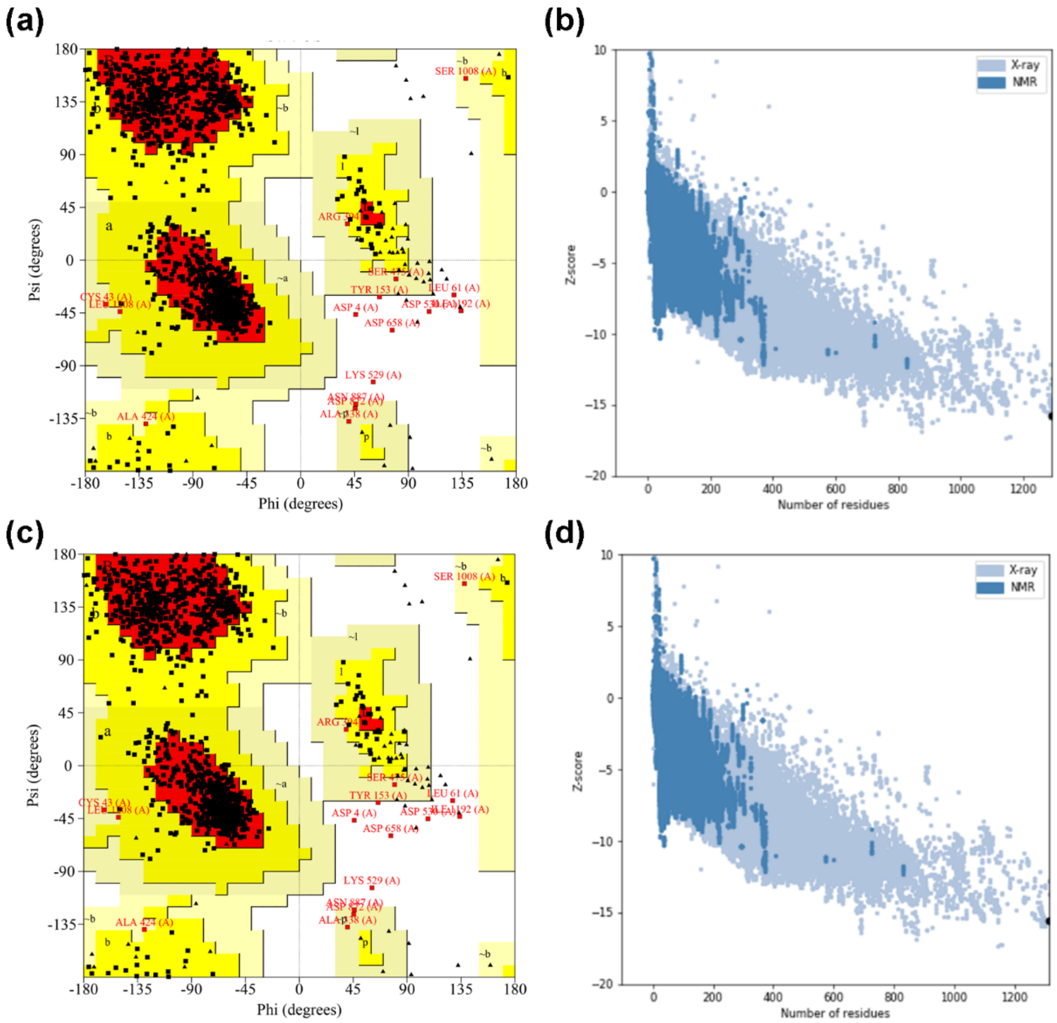

Figure S7. Evaluation results of Ramachandran (a, c) and Z-score distribution (b, d) plots of the original (a, b) and repaired (c, d) XO proteins.
